# Progress in Medicine

**Published:** 1898-10-22

**Authors:** 


### Progress in Medicine.

### DISEASES OF DIGESTIVE ORGANS.

(*Continued from page* 50.)

With reference to the value of laparotomy in tuberculous peritonitis, Lauder Brunton[41] suggested that it is of benefit in some cases of pulmonary disease, and mentioned an instance where great benefit followed not only to the peritoneal, but also to the lung disease. He thought that possibly the air acting on the peritoneum caused the manufacture of anti-toxin on a large scale. As a means of diagnosis showing the exact size of organs in direct contact with the body wall, Maguire[42] again draws attention to the use of auscultatory percussion, and of simple palpation such as is used over the eye in glaucoma. The former method does not, indeed, give the true size of the heart when it is overlaid by lung, nor of the stomach when overlaid by the colon; but it enables us to map out exactly the size of the colon, and of the tissues and lobes of the lungs. It shows the upper limits of a pleural effusion, and enables us to compare the height of the apices of the two lungs. Simple palpation will, with a little practice—astonishing as it may seem—show more accurately than any other method the size of the heart, spleen, and liver. Even through the sternum the resistance of the heart can be felt by pressure with a single finger at various points.

**The Colon.**—Treves recently expressed the view that "idiopathic dilatation of the colon" in young children is due to congenital defects in the bowel, with mechanical obstruction, though he recognised most fully the fact that distension of any part of the alimentary canal may take place without obstruction, as for instance, the ballooning of the rectum, dilatation of the stomach, or that of the intestines in marked peritonitis. Cheadle[43] points out that this statement is too absolute, and that cases of long-continued dilatation of the colon do occur in children without any stricture, and accompanied by chronic constipation. David B. Lees[44] brought forward before the Clinical Society two cases of intussusception. In one the cæcum, the valve, and an inch of the ileum had passed down the colon. Partial reduction was effected more than once by injections of water under anæsthetics, but finally laparotomy and excision of the part resulted in a cure. In the second, complete reduction was effected by irrigation and the use of an ice bag for eight days to reduce the swelling of the cæcum. In acute cases he thought this method would be useless from the tendency to collapse, but, on the other hand, peritoneal operations in young infants are attended with great risk. Barker favoured immediate operation in all ordinary cases, because there are many where other treatment is hopeless, and delay may result in gangrene.

**Thrombosis of Abdominal Vessels.**—Köster[45] gives details of three cases of thrombosis of the mesenteric veins, showing that the diagnosis cannot be made with certainty during life, as the symptoms are the same as those seen in acute obstruction and perforative peritonitis. He gives also a case of embolism of the mesenteric artery, and one of thrombus in the splenic vein. Rose Bradford[46] reported an instance of obstruction caused by thrombosis of the superior mesenteric vein. Pain, vomiting, and at first, diarrhœa, with finally a mass in the left iliac region were noticed, but the true condition was discovered too late by laparotomy. Other cases were referred to at the Clinical Society when this one was brought up.

**Dysentery.**—H. P. Harris[47] has written an elaborate monograph on the amœbic form with an analysis of 35 cases under his care, of which 12 recovered, four improved, and nine died, while 10 passed away from observation. All complained of griping pains with the passage of stools from two to 40 times in the 24 hours. Liver abscess, piles, peritonitis, and appendicitis were noted among the complications. He finds that the disease generally arises in filthy conditions, especially when the water supplies and food are deficient, and that it most often attacks the male sex in adult life. Though the connection of the amœbæ with the disease is not absolutely made out, yet the evidence in favour of it is very strong. They stain in various ways, but perhaps the best results are obtained by hardening in corrosive sublimate, and then soaking in iron alum hæmatoxylin or methylene blue. Ulceration of the intestines is very common, and the cause of many of the complications; the ulcers spread either by changes in the submucous tissue, or in other cases by softening of the mucous edges without any undermining. The liver abscesses do not contain true pus, but broken down tissue, and there is no inflammatory zone around them.

When the disease has become chronic, the chances of recovery are very bad. All kinds of drugs have been employed. Injections of quinine, potassium permanganate, and of hydrogen dioxide have been tried with varying success. Harris found remarkable results at times with the last-mentioned, the improvement being striking and immediate. Commercial hydrogen dioxide is diluted from four to eight times with water, and a quart of this mixture injected, per rectum, twice daily. Salol, opium, and sulphuric acid have often been given by the mouth for this affection. F. Roemer[48] confesses that no diagnostic mark exists by which tropical dysentery can be distinguished from European. In both amœbæ can be found, but whether they are the pathological agent is uncertain. The strongest argument for their toxic power is seen in the fact that they and no other organisms are found in the débris of liver abscesses, and that injections of this débris produce typical dysentery in cats. T. Maberley,[49] who treated 100 cases of dysentery at the Cape, with only one death, by the use of *monsonia ovata*, reports good results in gastric and intestinal ulceration from the use of the tuberous-rooted pelargonium, closely allied to P. reniforme. He employed the monsonia also with apparent success in typhoid ulceration. He concludes that these plants are not poisonous, and that the monsonia is valuable for disease of the lower part of the intestinal tract, and the pelargonium for the stomach and small intestines. Two drachm doses of a tincture of these plants are given for dysentery, and a drachm in typhoid about the eighteenth day for two or three days. The drugs seem to have no value for ordinary diarrhœa. Buchanan[50] divides dysentery in India into three classes, mild, acute, and chronic. Rest in bed and low diet quickly cures the mild form. In strong persons suffering from the acute disease he prefers 20-24 grain doses of ipecacuanha, in weakly patients he gives a saturated solution of Epsom salts in two drachm doses three or four times a day. For chronic cases which resemble the amœbic form, but rarely cause liver abscesses, and occur in broken-down cachectic persons, salol, perchloride of mercury, with opium and other drugs, may be tried, but rest and a milk diet are all-important. He does not regard the amœba as the active agent. A. Schmidt[51] has investigated the various forms of mucus found in the stools in different diseases. He says that mucin and not fibrin is the basis of all these membranous discharges. For the diagnosis of ulceration the absence of mucus among the cells is more important than the number of sound cells. Mucus very rarely passes undissolved from the small intestine to the anus; generally the nuclei are alone left when voided.

[41] Brit. Med. Jour., Mar. 5. [42] Brit. Med. Jour., Feb. 19. [43] Lancet, Feb. 5. [44] Lancet, May 21. [45] Brit. Med. Jour., July 2. [46] Lancet, April 30. [47] Amer. Jour. Med. Sci., April. [48] Indian Med. Rec., Mar. 1. [49] Lancet, July 16. [50] Lancet, July 2. [51] Amer. Jour. Med. Sci., Mar.

